# SpyGlass-Guided Photodynamic Therapy for Unresectable Cholangiocarcinoma: A Case Report and Review of the Literature

**DOI:** 10.3389/fonc.2022.890735

**Published:** 2022-08-10

**Authors:** Jiangjiao Zhou, Li Xiong, Wei Liu, Chun Liu, Chao He, Heng Zou, Yu Wen, Liangwu Lin

**Affiliations:** ^1^ Department of General Surgery, The Second Xiangya Hospital, Central South University, Changsha, China; ^2^ Powder Metallurgy Research Insititute, Central South University, Changsha, China

**Keywords:** Case report, cholangiocarcinoma, ERCP, photodynamic therapy, SpyGlass

## Abstract

Endoscopic retrograde cholangiopancreatography (ERCP) and biliary stent placement are standard palliative care procedures for patients with unresectable cholangiocarcinoma. However, the bile duct mucosa cannot be observed directly during the placement of a light diffuser in photodynamic therapy (PDT) and biopsy. SpyGlass can solve the above two problems. In this paper, we report the case of a patient who presented with obstructive jaundice and underwent endobiliary stenting placement several times. However, cholangiocarcinoma hyperplasia still led to stent blockage. We applied SpyGlass to guide the accurate placement of a light diffuser and evaluated the tumor necrosis after PDT. Results revealed the good application effect of this device.

## Introduction

Endoscopic retrograde cholangiopancreatography (ERCP) and biliary stent placement are standard palliative care procedures for patients with unresectable cholangiocarcinoma. Several studies reported the survival benefits of photodynamic therapy (PDT) in conjunction with endobiliary stenting (1–3). However, the bile duct mucosa cannot be observed directly during ERCP, compromising the accurate irradiation of the focus during PDT. Moreover, obtaining tissue samples from intrabile duct tumors, especially hilar bile duct tumors, for subsequent radiotherapy and chemotherapy is complicated. SpyGlass allows the direct observation of the bile duct mucosa and guidance of light diffuser placement while obtaining tumor tissue, thereby solving the two problems mentioned above. It can also be used to evaluate directly the therapeutic effect of PDT.

In this paper, we report the case of a patient with distal cholangiocarcinoma. The patient presented with obstructive jaundice and refused surgical treatment. Then, biliary stents were placed in this patient several times under ERCP. However, cholangiocarcinoma hyperplasia still led to stent blockage. In PDT, we applied SpyGlass to guide the placement of a light diffuser and evaluated the tumor necrosis after PDT. Results revealed the good application effect of SpyGlass.

## Case Report

**Figure 1:**
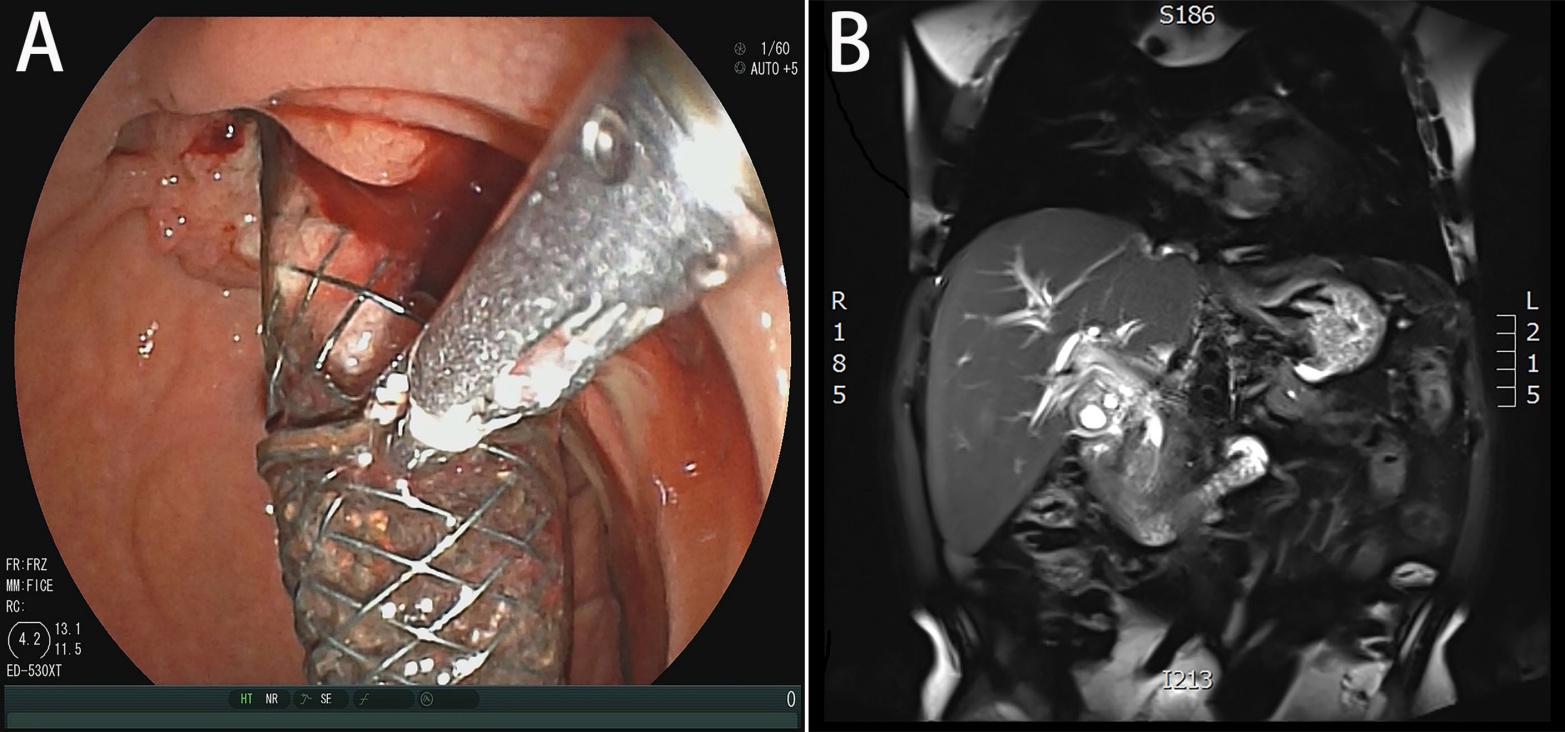
**(A)** MRCP demonstrated that neoplasm was in the distal common bile duct. **(B)** Duodenoscopy showed that the biliary stent was filled and blocked by tumor tissue and bile mud.

**Figure 2:**
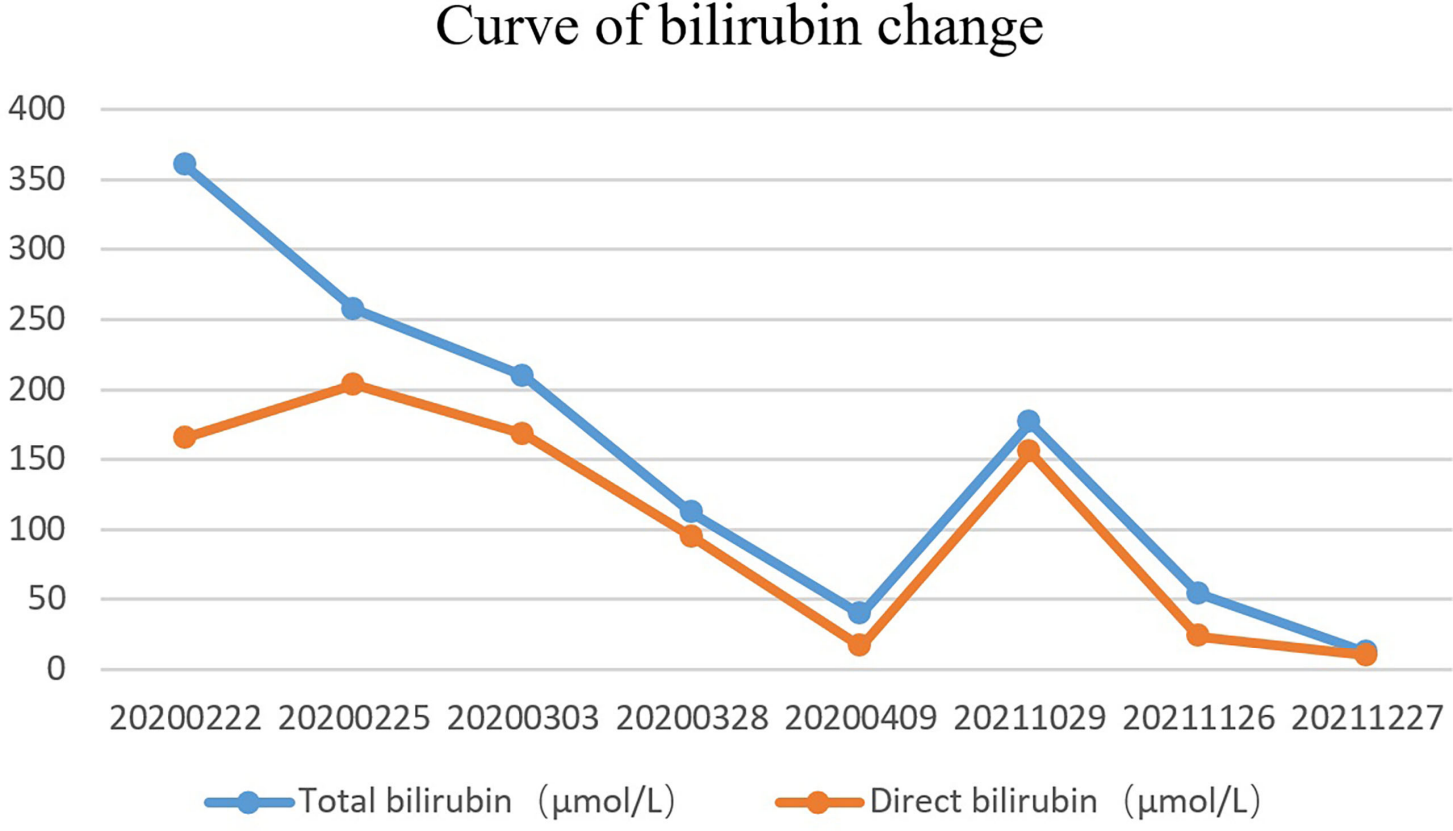
Curve of bilirubin change.

A 51-year-old married male visited the local hospital because of jaundice. Magnetic resonance cholangiopancreatography (MRCP) revealed that the intrahepatic and extrahepatic bile ducts were dilated and lesions were located at the distal common bile duct. Endoscopic nasobiliary drainage (ENBD) was performed, biopsy showed papillary dysplasia and canceration of bile duct epithelium (well differentiated adenocarcinoma). The decrease in serum bilirubin was not obvious more than 10 days after ENBD, and then the patient visited our hospital for the first time on February 21, 2020. Blood test results showed that the total bilirubin was 360.2 µmol/L and the direct bilirubin was 286.1 µmol/L. Redoing MRCP suggested that neoplasm was in the distal common bile duct, with a size of approximately 19 mm×16 mm×30 mm (**Figure 1A**) and common hepatic artery invasion. The intrahepatic and extrahepatic bile ducts were obviously dilated, and the pancreatic duct was not dilated and exported separately in the duodenal accessory papilla. No liver and retroperitoneal lymph node metastasis. The diagnosis was carcinoma of the distal common bile duct T_4_N_X_M_0_ (According to the 8th edition of the American Joint Committee on Cancer). The patient underwent percutaneous transhepatic biliary drainage (PTCD) and was discharged on March 5, 2020. After discharge, the PTCD drainage tube drained approximately 800 mL of bile every day, and the serum bilirubin gradually decreased. Due to common hepatic artery invasion, the patient refused surgical treatment and required PTCD tube movement. Thus, he was hospitalized again on March 26, 2020. Blood test showed that the total serum bilirubin was 111.9 µmol/L, and the direct bilirubin was 94.5 µmol/L. ERCP was performed on April 2, 2020 for biliary metal covered stent placement, and the PTCD tube was removed. After discharge, the patient refused to receive further treatment. The patient developed jaundice again after 16 months and was hospitalized on October 29, 2021. ERCP showed that the biliary stent was filled and blocked by tumor tissue and bile mud (**Figure 1B**). Thus, the biliary metal stent was removed, and double plastic stents were placed into the left and right hepatic ducts. Patients were willing to accept PDT with our advice. Photofrin (2 mg/kg) was administered intravenously 48 h prior to PDT. ERCP and SpyGlass were employed on November 26, 2021. Cholangiocarcinoma was located under direct vision, and bile duct tumor biopsy was performed (**Supplementary Video 1**). The pathological result was highly differentiated adenocarcinoma. Then, a 4 cm-long, cylindrical light diffuser and sleeve were inserted, and photoactivation was performed at 630 nm with a light dose of 180 J/cm^2^ and irradiation time of approximately 750 s. A plastic stent was placed after PDT. The next day, SpyGlass was rechecked (**Supplementary Video 2**). Results showed that most of the tumor tissues had ischemic necrosis changes, and a few tumor tissues indicated poor ischemic necrosis at the uppermost edge of the tumor. The guide wire was placed into the catheter and light diffuser, and the upper end of the bile duct was treated with PDT. After the treatment, a plastic stent was placed. One month later, SpyGlass was performed and revealed that most of the tumors were necrotic (**Supplementary Video 3**). The necrotic tissue was cleaned up and for rapid pathological diagnosis, it was infiltrated by numerous inflammatory cells and no adenocarcinoma cells were observed. Then the plastic stent was retained. Up to now, the patient has no jaundice (**Figure 2**) and has good spirit and physical strength.

## Discussion

A consensus on whether or not PDT can benefit the survival of patients with unresectable cholangiocarcinoma has yet to be reached, and high-level evidence-based medical evidence is lacking. However, most studies still suggested that PDT could prolong the survival of patients (2). Survival in one meta-analysis by Moole et al. in patients with cholangiocarcinoma showed a median survival benefit of 7.6 months after PDT (3). Haider et al. demonstrated that PDT provides a significant survival benefit of almost 14 months (1). By contrast, Stephen et al. reported that 13 (28%) PDT plus stent patients and 24 (52%) stent-alone patients received subsequent palliative chemotherapy. After a median follow-up of 8.4 months, overall survival and progress-free survival were worse in the patients who received PDT than in those who received the stent alone (4). In the present case, results showed that PDT is valuable in the treatment of unresectable cholangiocarcinoma. For this case, we only wanted to drain the biliary tract by endobiliary stenting at the beginning. However, with the simple placement of stents, the tumor can grow into the stents and lead to blockage. PDT can lead to obvious necrosis and abscission of the tumor tissue in the bile duct, which is important in maintaining the patency of the bile duct to prolong the survival time and improve the quality of life of patients.

For ERCP-guided PDT of cholangiocarcinoma, the lesion can be located and irradiated by cholangiography. However, cholangiography for cholangiocarcinoma depends on indirect signs, which are sometimes inaccurate. Under the direct vision of SpyGlass, the tumor location and light diffuser placement are undoubtedly more accurate. Such accuracy is important for the intraductal papillary mucinous neoplasm (IPMN) of the biliary and pancreatic ducts. IPMN is a borderline tumor. Previous studies have suggested that PDT has a good therapeutic effect (5–7). However, the intraductal papillary tumor is hard to locate in PDT. The pancreaticobiliary duct is filled with mucus; thus, only indirect signs of ERCP are used to locate the papillary tumor (8, 9). SpyGlass has great advantages, but its use in patients with IPMN has yet to be reported. For cholangiocarcinoma, since 2011, many scholars have attempted to treat cholangiocarcinoma with SpyGlass-guided PDT, which shows effectiveness and safety (10, 11). SpyGlass can also be used to evaluate the effect of PDT (11). Our case also achieved good therapeutic effect.

ERCP has become a key technology in the diagnosis and treatment of biliary diseases, but it cannot be used to observe the biliary mucosa directly and perform biopsy, which limit its applications. During hepatobiliary surgery, choledochoscopy, T-tube sinus choledochoscopy, and percutaneous transhepatic choledochoscopy can be used to observe the biliary mucosa directly, but these procedures are invasive. SpyGlass allows the direct observation of the bile duct mucosa. It is simple and convenient to operate by one person and can be operated in a normal bile duct. Thus, SpyGlass-guided PDT has unique advantages for unresectable cholangiocarcinoma, especially for IPMN. However, the application of SpyGlass also has some disadvantages. For instance, the camera is easy to be damaged during operation, and the duodenal papilla must be greatly cut or expanded. Direct oral choledochoscopy can only be performed in cases with bile duct diameter > 8 mm. Thus, direct oral choledochoscopy is difficult to perform in patients with diffuse bile duct stenosis, and complications such as cholangitis and gas embolism can still arise (12, 13). We are optimistic about the application of SpyGlass-guided PDT for unresectable cholangiocarcinoma, especially IPMN. We look forward to selecting appropriate cases to take the lead in the near future.

Illustration

## Data Availability Statement

The raw data supporting the conclusions of this article will be made available by the authors, without undue reservation.

## Ethics Statement

Written informed consent was obtained from the individual(s) for the publication of any potentially identifiable images or data included in this article.

## Author Contributions

LX and JJZ took the lead in drafting the manuscript. CL, WL, and CH performed SpyGlass-guided photodynamic therapy. LWL, HZ, and YW provided supervision and participated in the literature review and in drafting the manuscript. All authors have read and approved the final manuscript.

## Funding

This work was support by Changsha Municipal Natural Science Foundation (NO. kq2014245) and Natural Science Foundation of Hunan Province (NO. 2021JJ70138).

## Conflict of Interest

The authors declare that the research was conducted in the absence of any commercial or financial relationships that could be construed as a potential conflict of interest.

## Publisher’s Note

All claims expressed in this article are solely those of the authors and do not necessarily represent those of their affiliated organizations, or those of the publisher, the editors and the reviewers. Any product that may be evaluated in this article, or claim that may be made by its manufacturer, is not guaranteed or endorsed by the publisher.

## Supplementary Material

Supplementary Video 1 Bile duct mucosa under direct vision *via* SpyGlass before PDT.

Supplementary Video 2 Bile duct mucosa under direct vision *via* SpyGlass 24 h after PDT.

Supplementary Video 3 Bile duct mucosa under direct vision *via* SpyGlass 1 month after PDT.
